# Supplementary material for: The mechanism of replication stalling and recovery within repetitive DNA
Source: Nat Commun. 2022 Jul 19;13:3953. doi: 10.1038/s41467-022-31657-x (PMC9296464; doi:10.1038/s41467-022-31657-x)

Uncropped gels Figures 1-6

Figure 1B

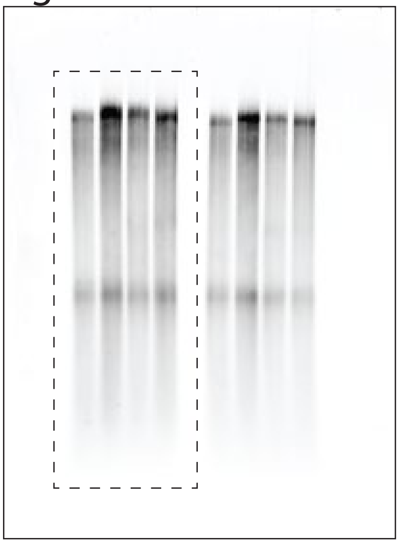

Figure 1C

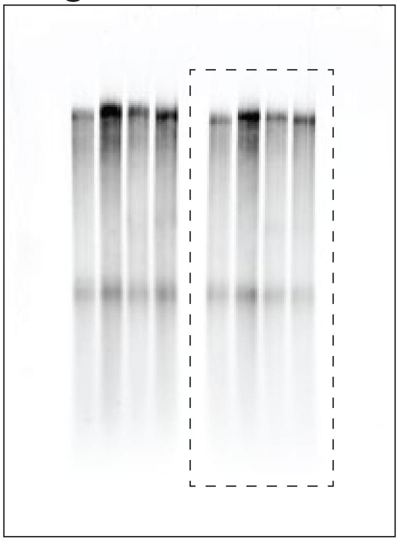

Figure 1D

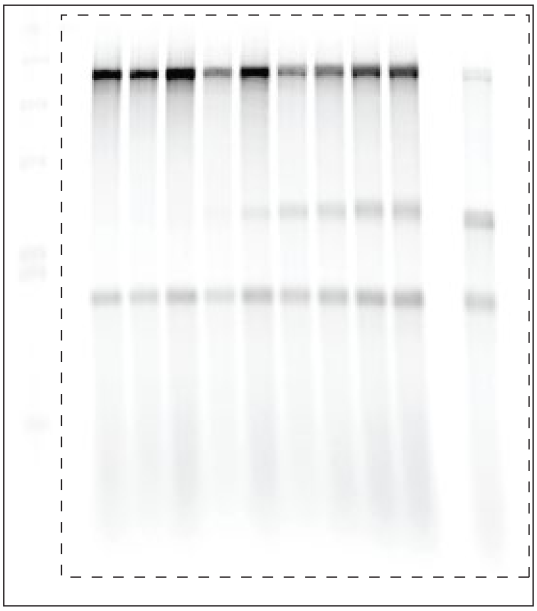

Figure 1E

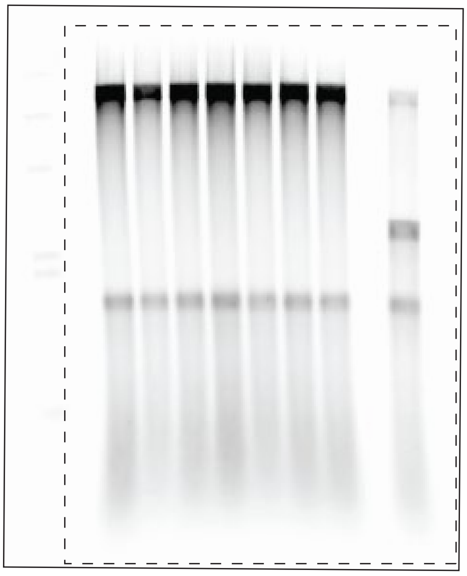

Figure 2A

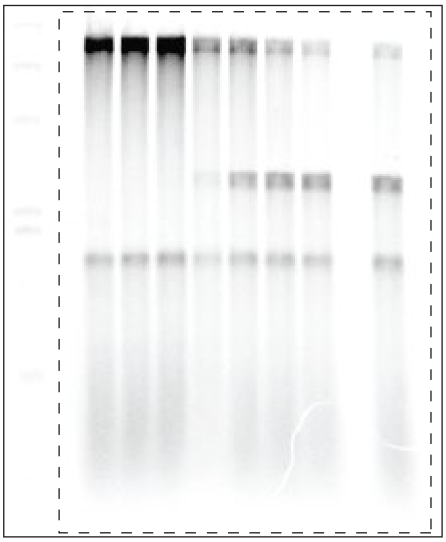

Figure 2B

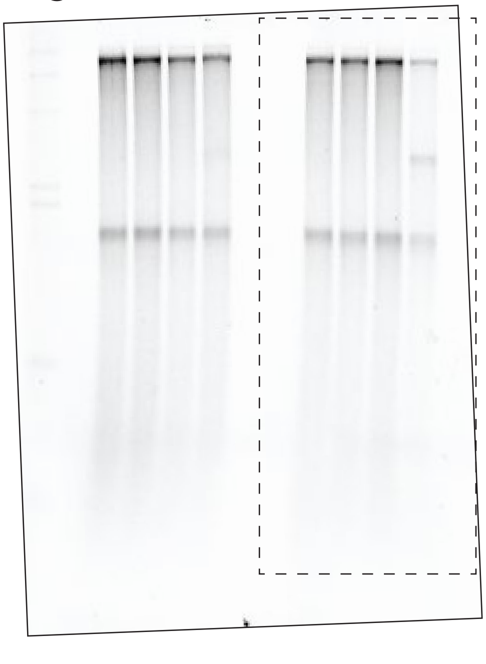

Figure 2C

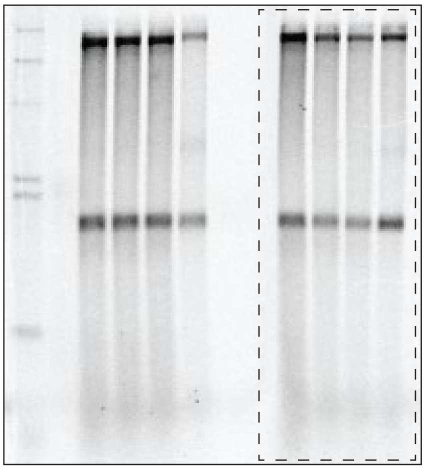

Figure 2D

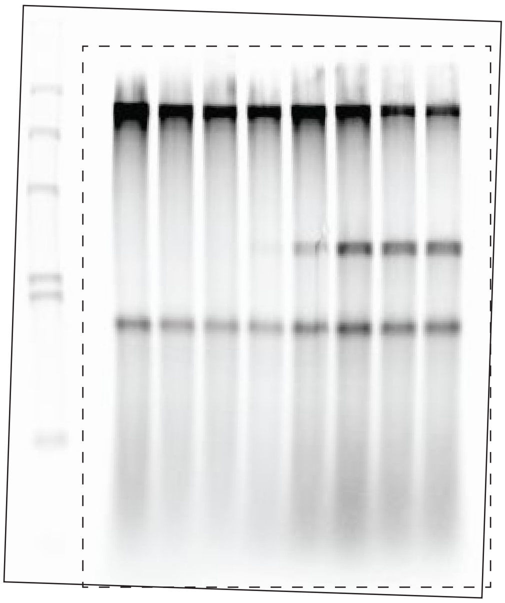

Figure 2E

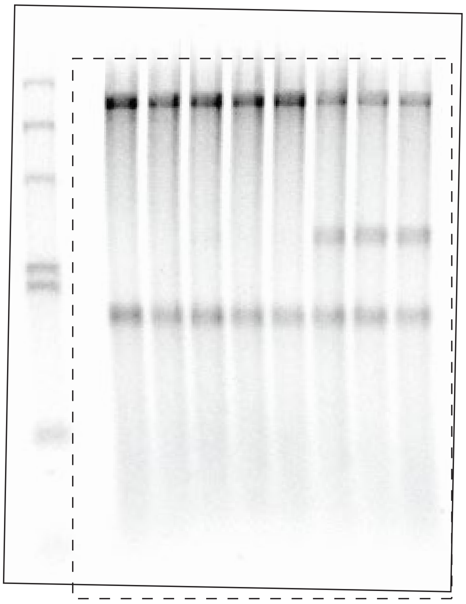

Figure 2F

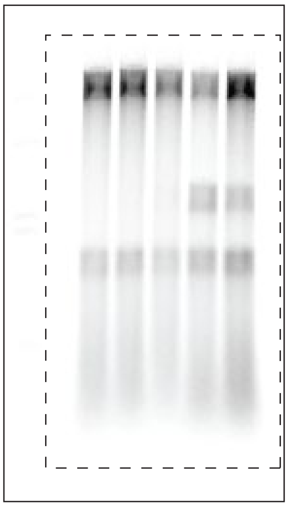

Figure 3A

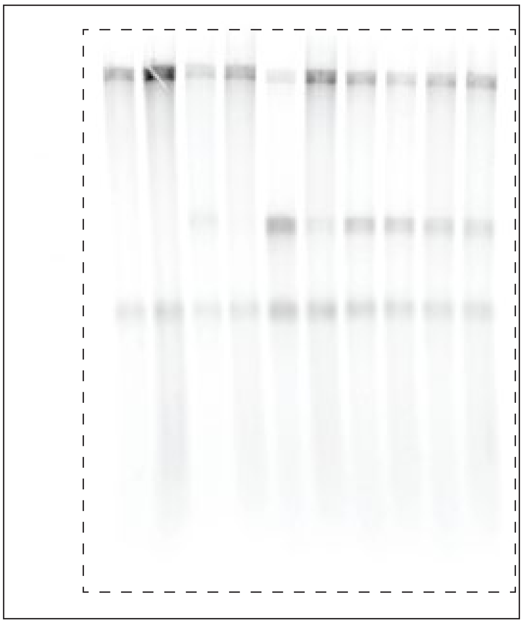

Figure 3B

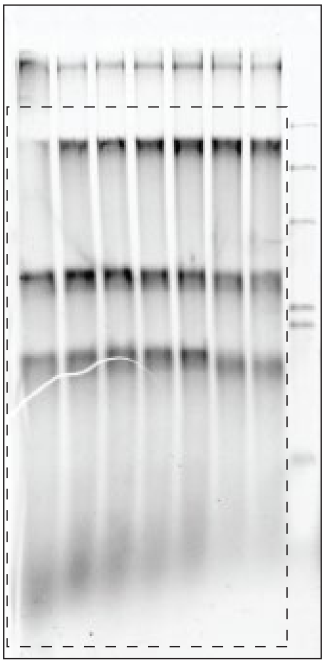

Figure 3C

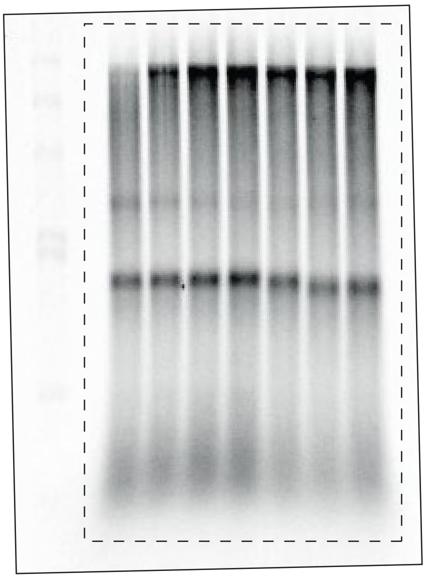

Figure 3D

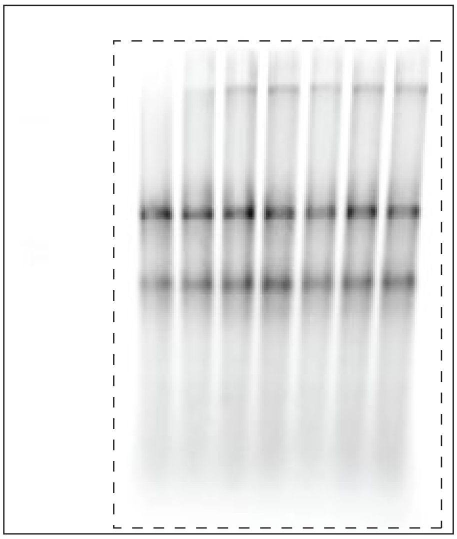

Figure 3E

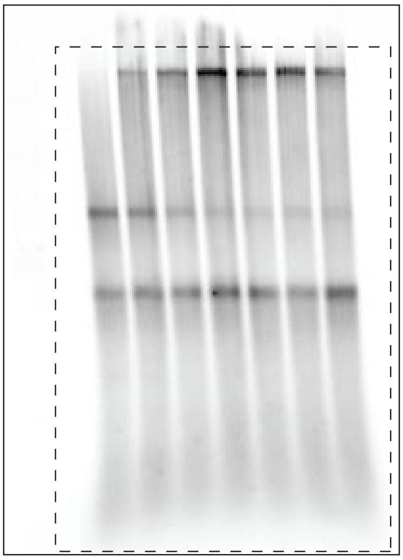

Figure 3F

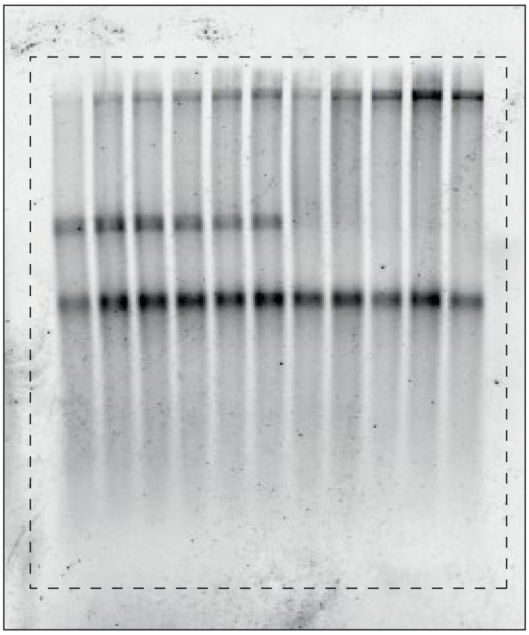

Figure 4A

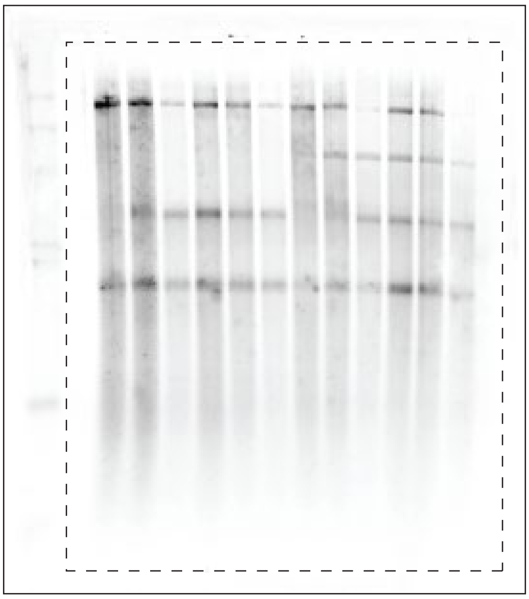

Figure 4B

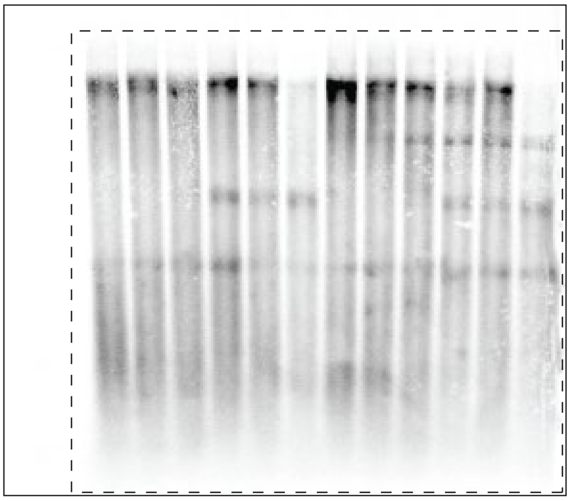

Figure 4C

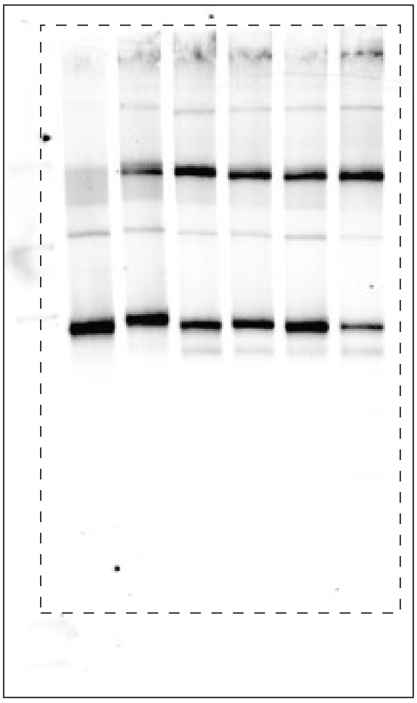

Figure 4D

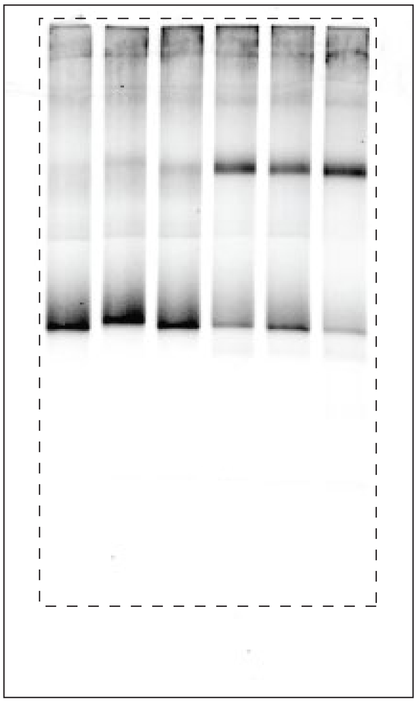

Figure 5A

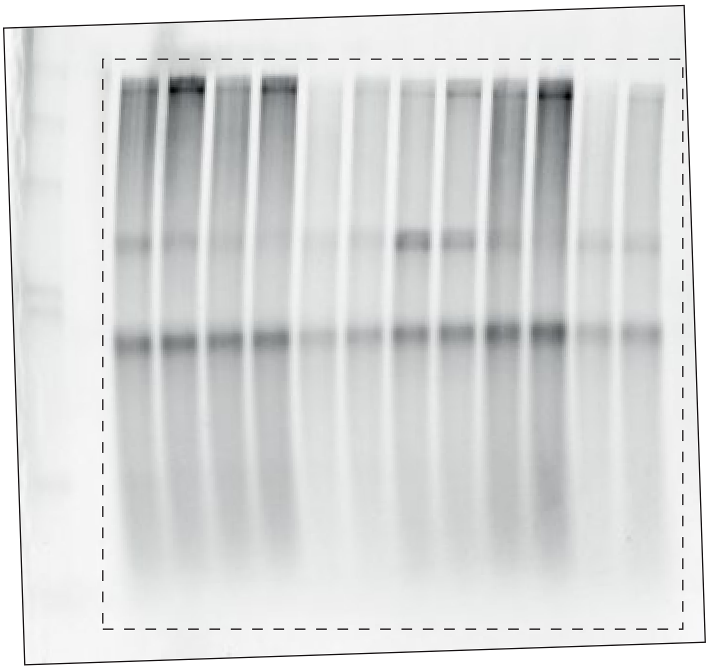

Figure 5B (left panel)

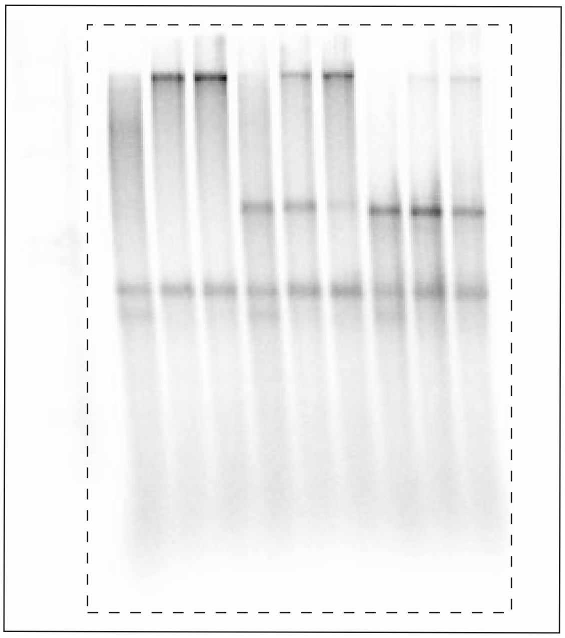

Figure 5B (right panel)

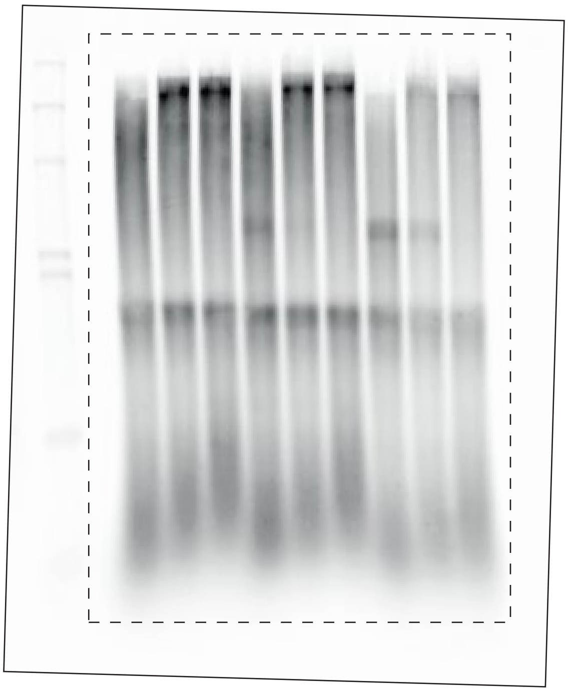

Figure 6A (left panel)

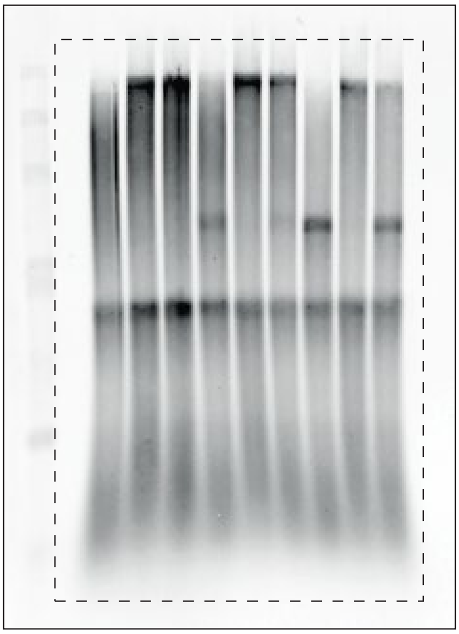

Figure 6A (right panel)

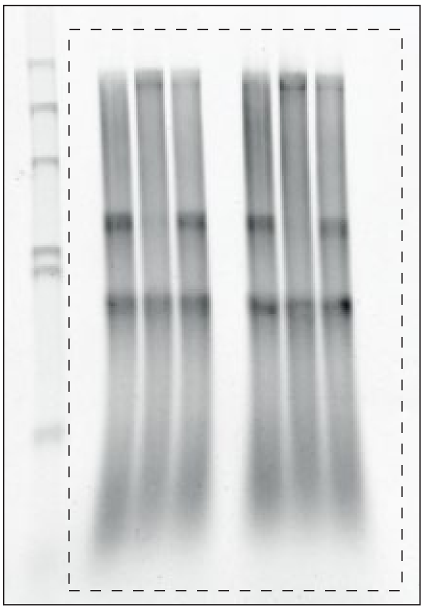

Figure 6B (left panel)

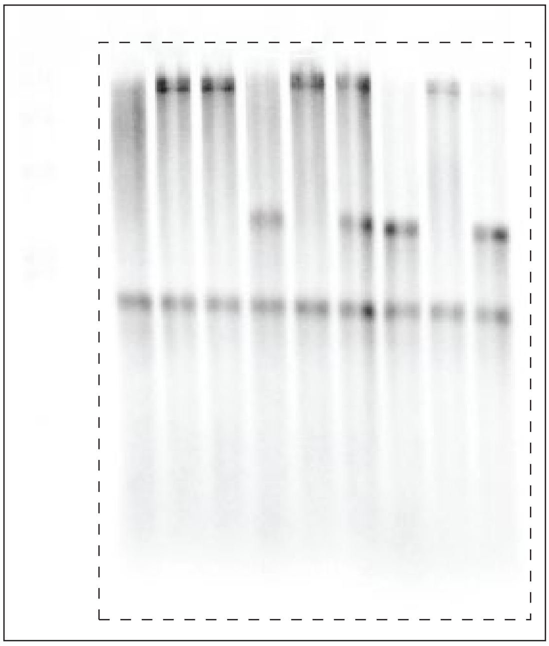

Figure 6B (right panel)

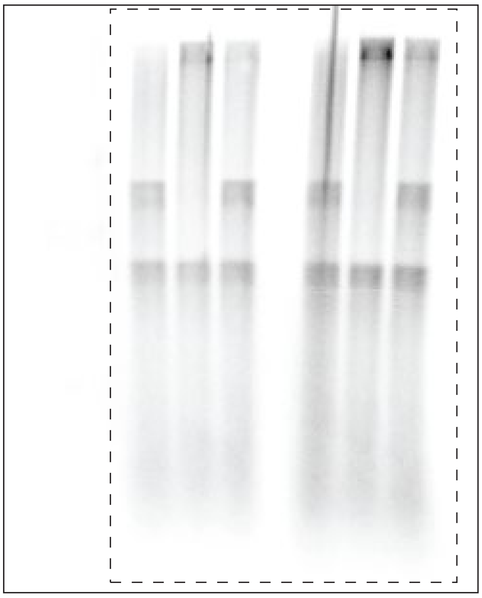

Uncropped gels Supplementary Figures 1-9

Fig. S1A

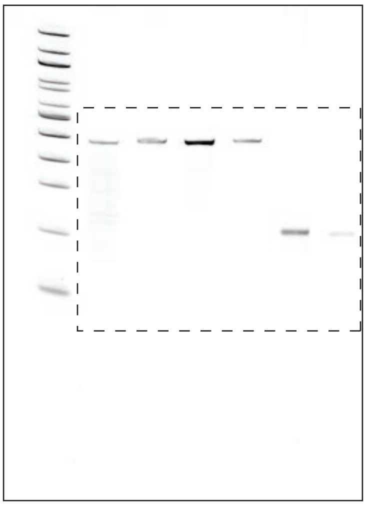

Fig. S1B

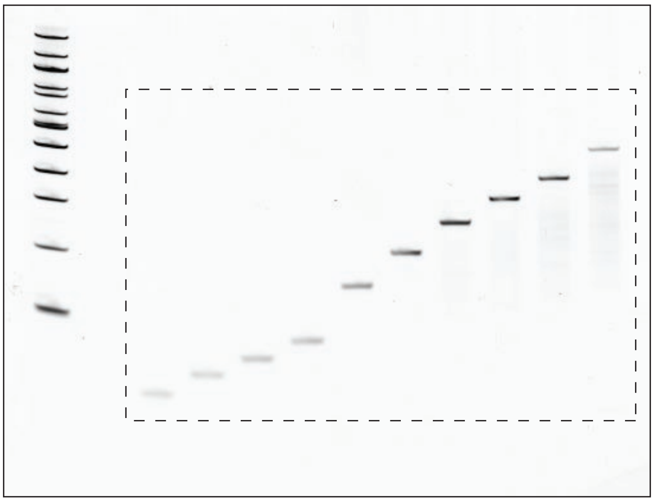

Fig. S1C

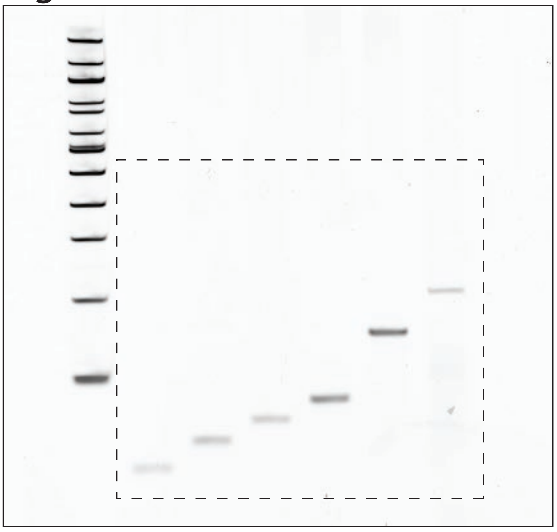

Fig. S1F

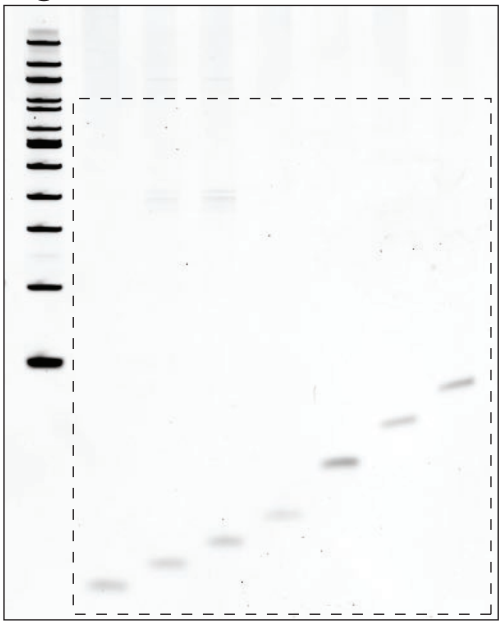

Fig. S1D Fig. S1E

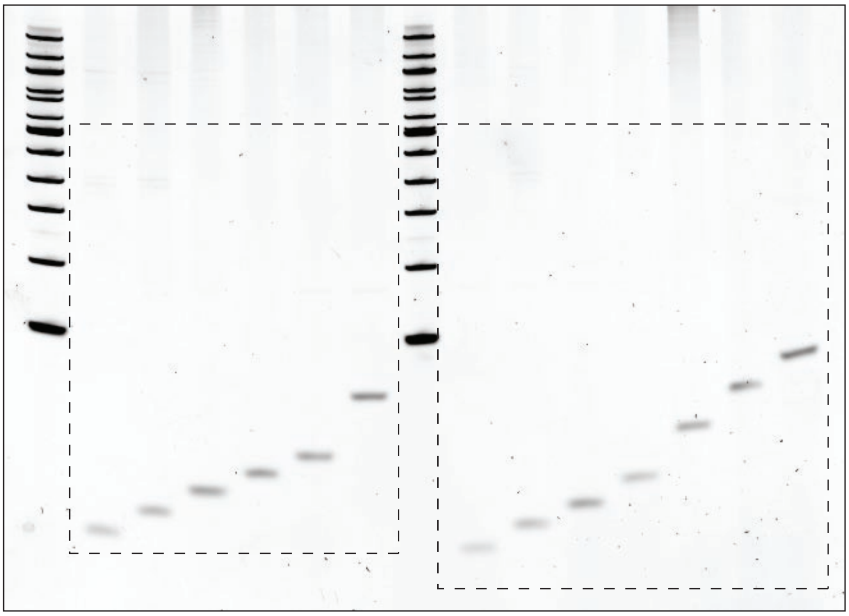

Fig S2B

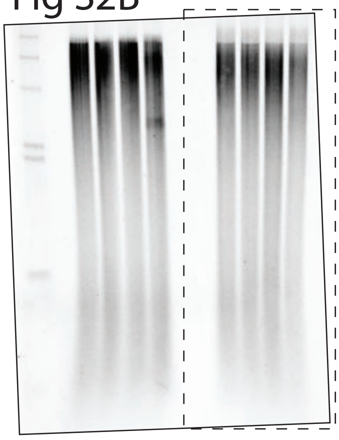

Fig S2C

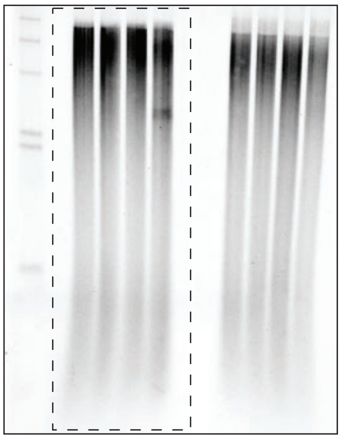

Fig S2D

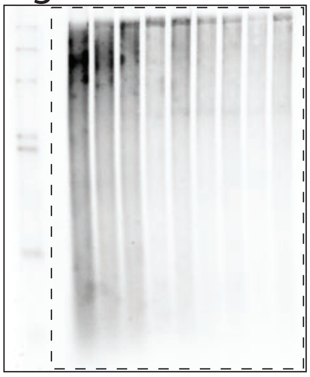

Fig S2E

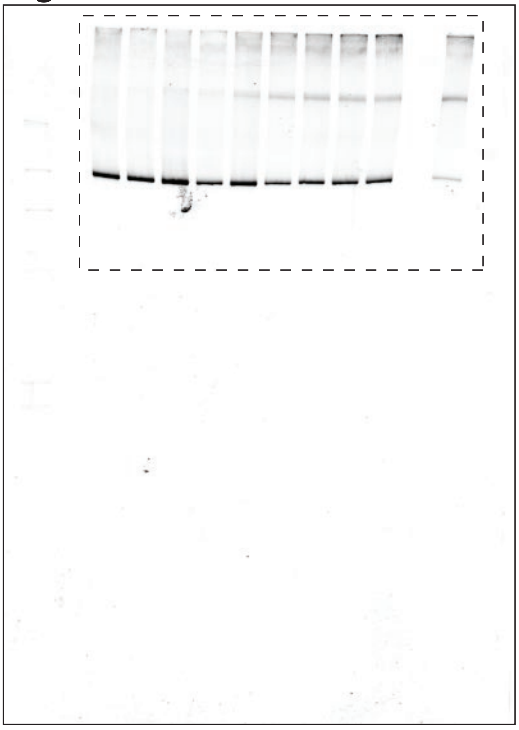

Fig S2F

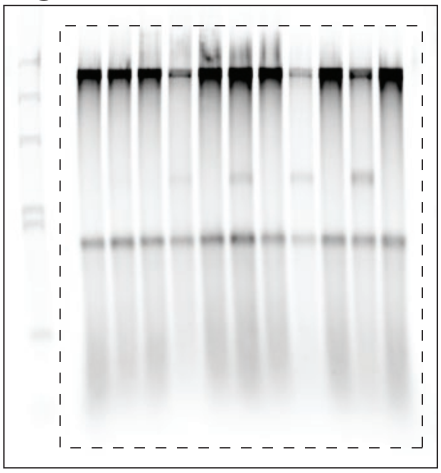

Fig S2G

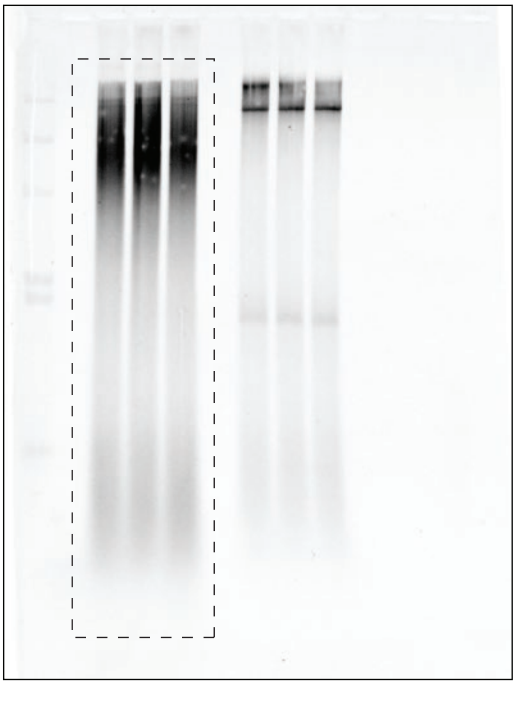

Fig S2H

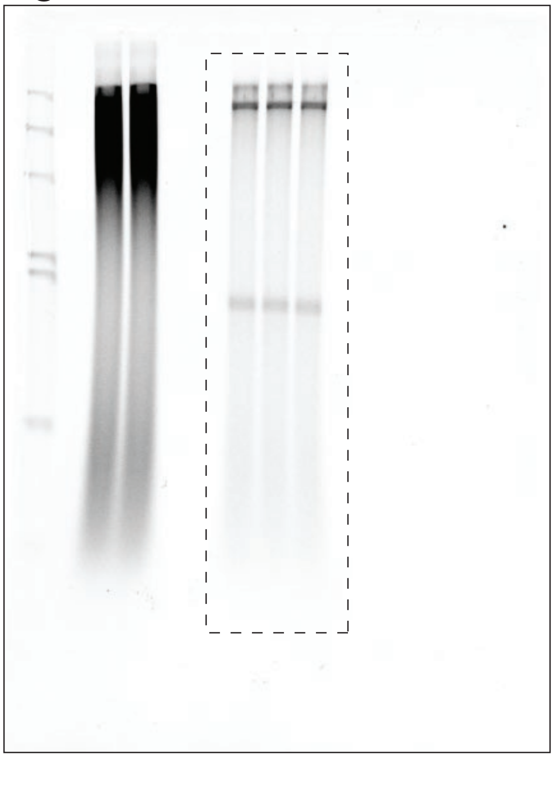

Fig S3A

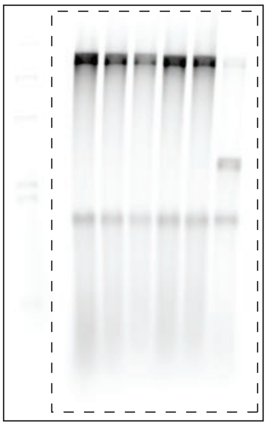

Fig S3B

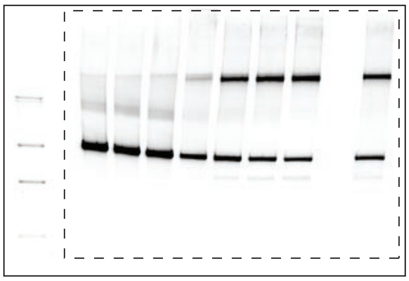

Fig S3C

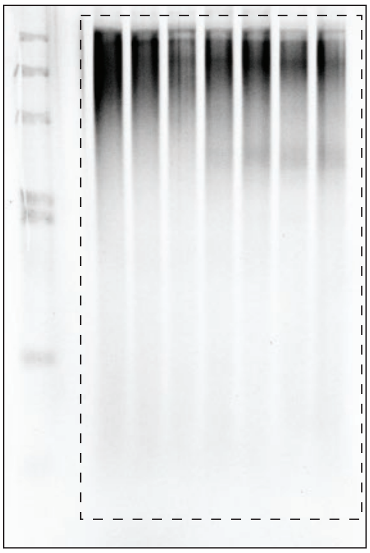

Fig S3D

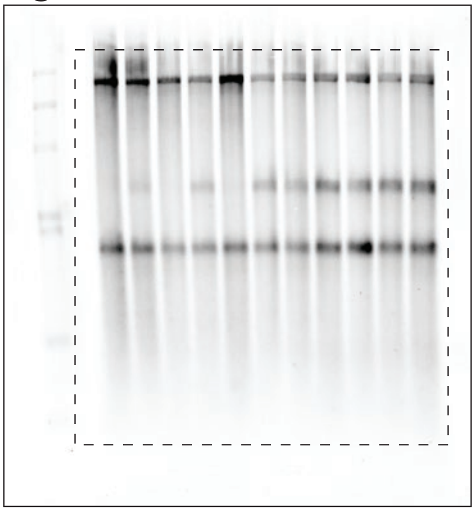

Fig S3E

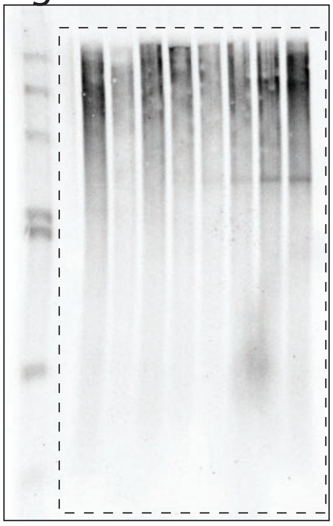

Fig S3F

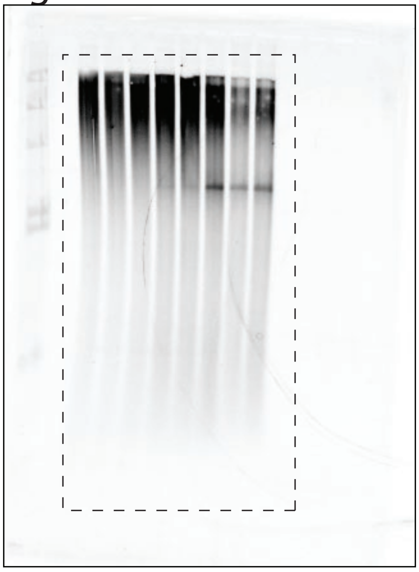

Fig S3G

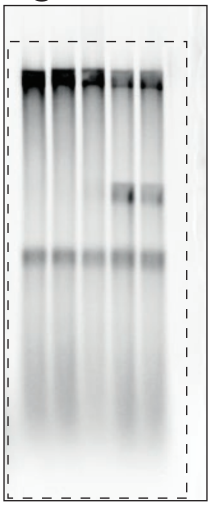

Fig S4A

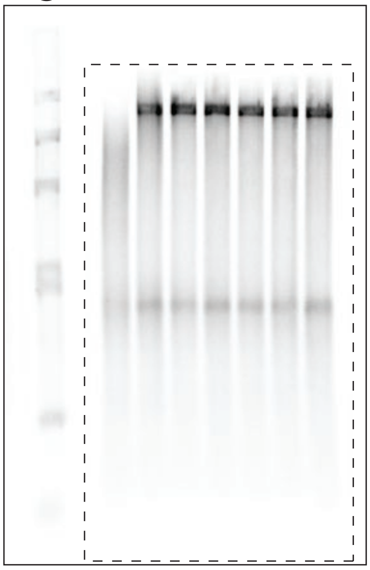

Fig S4B

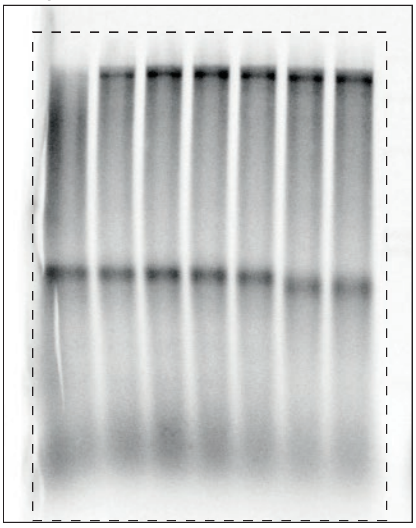

Fig S4C

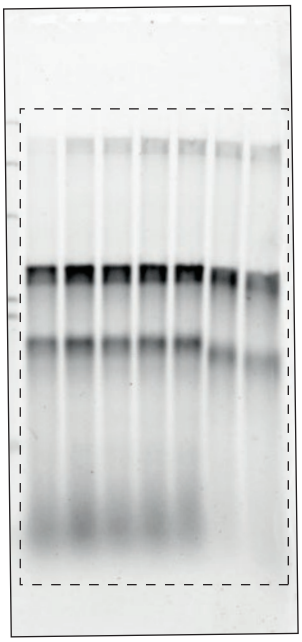

Fig S4D

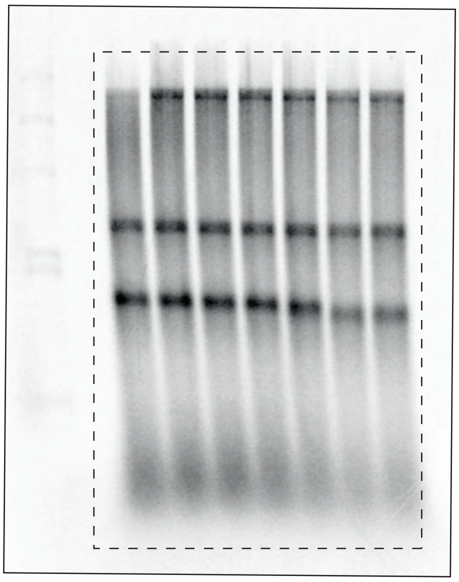

Fig S4E

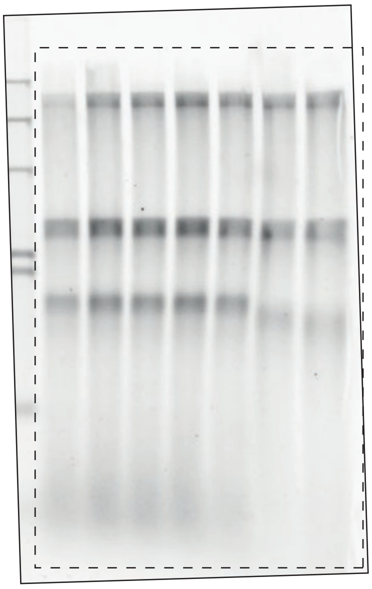

Fig S4F

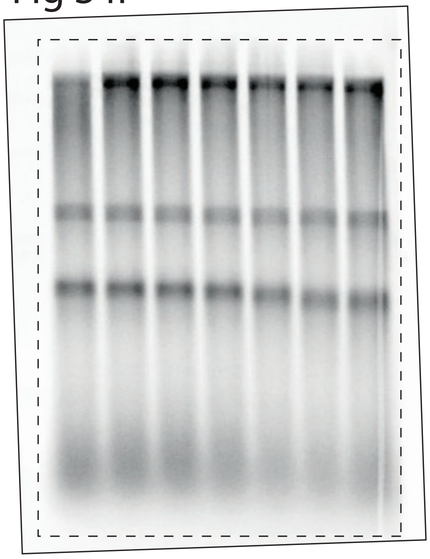

Fig S5A

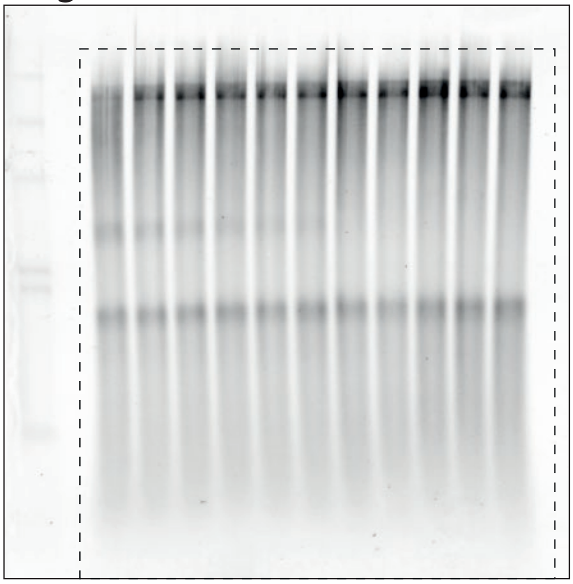

Fig S5B

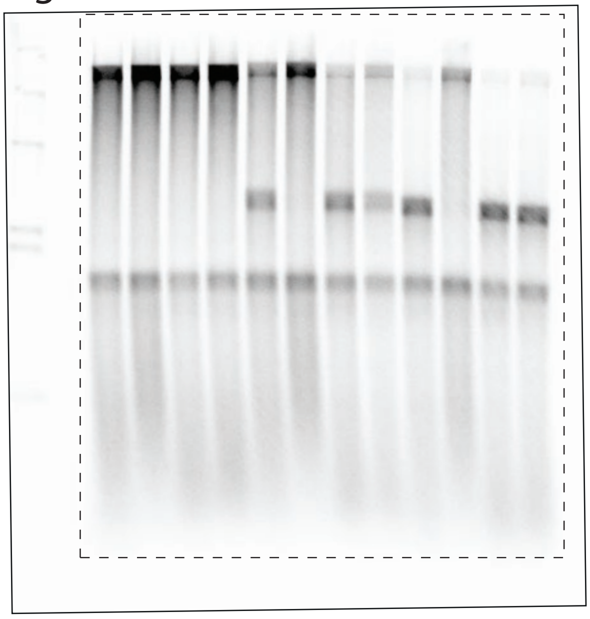

Fig S6A (left panel)

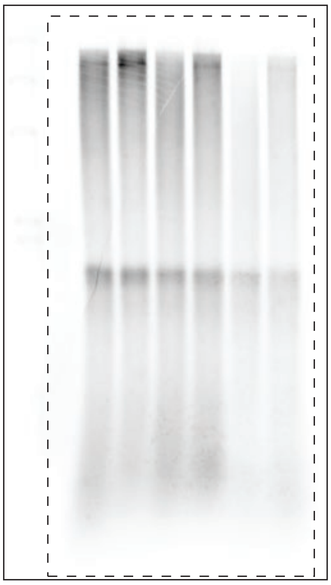

Fig S6A (right panel)

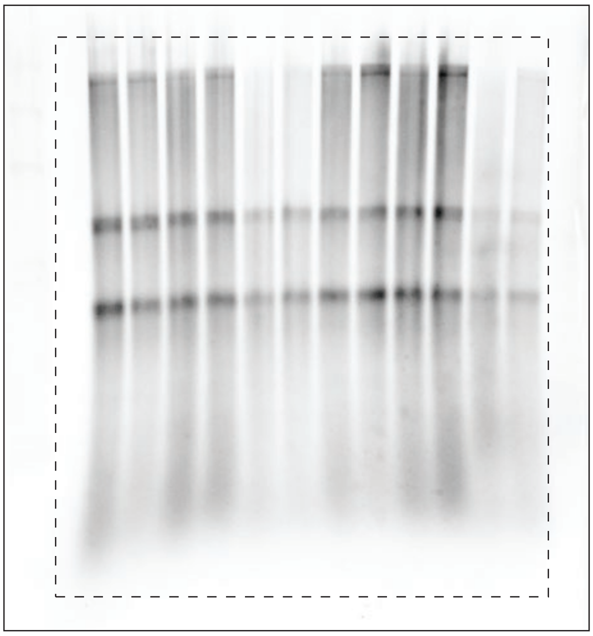

Fig S6B (left panel)

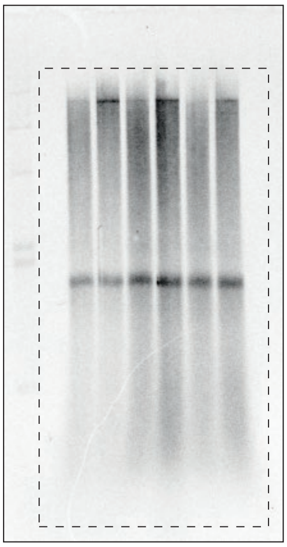

Fig S6B (middle panel)

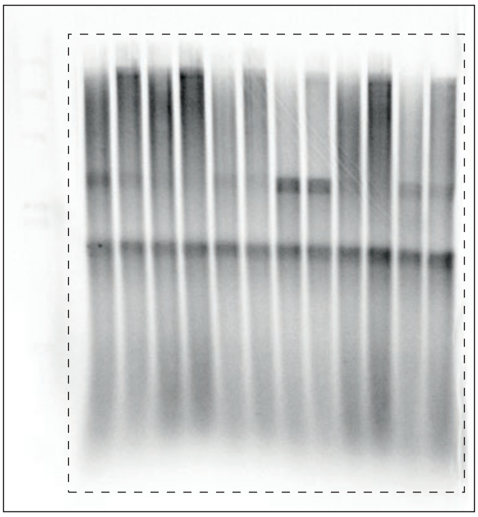

Fig S6B (right panel)

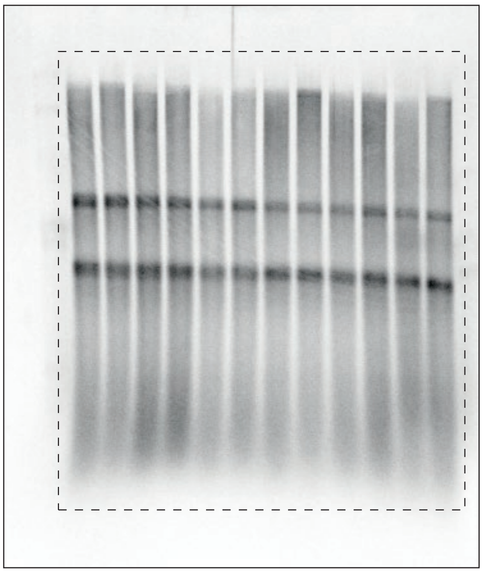

Fig S6C

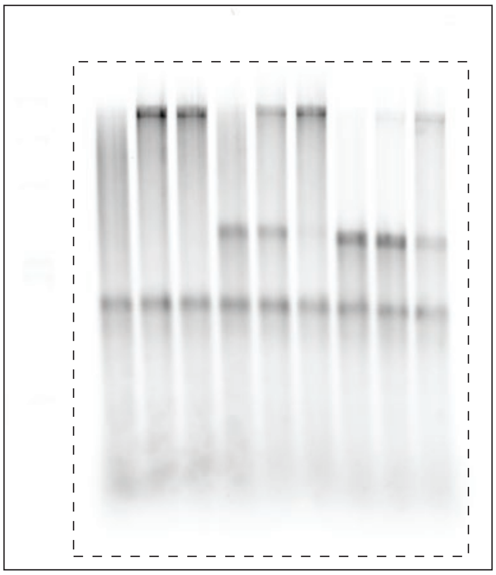

Fig S6D

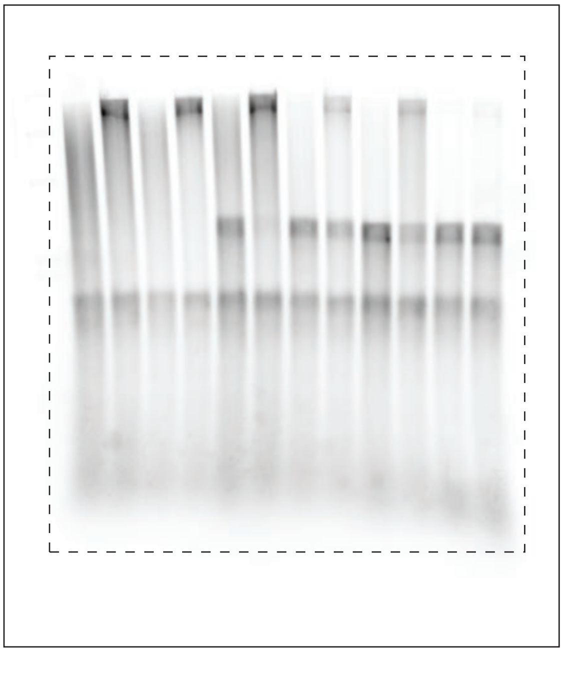

Fig S7A (left panel)

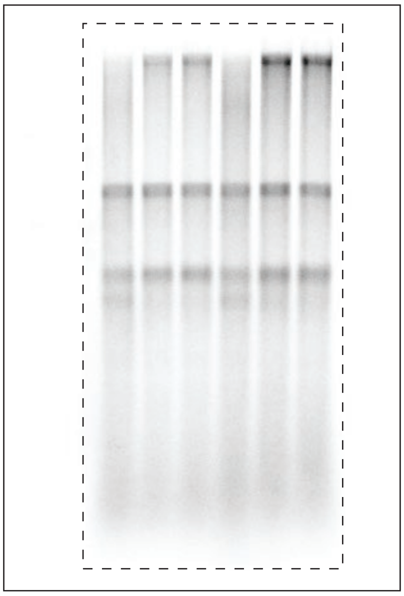

Fig S7A (right panel)

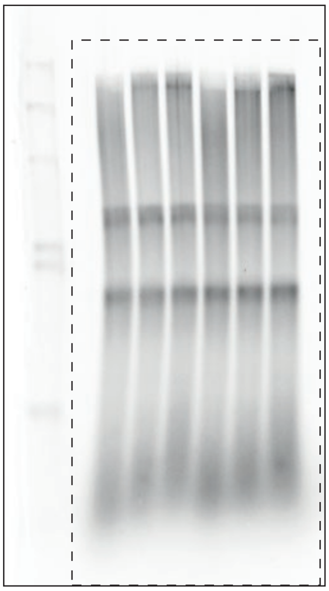

Fig S7B (left panel)

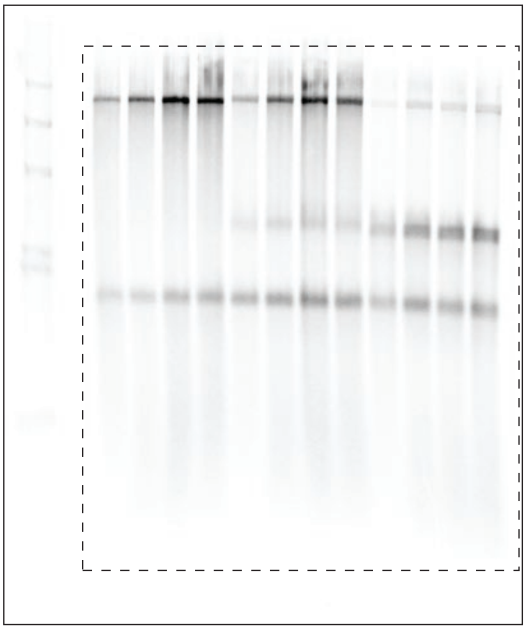

Fig S7B (right panel)

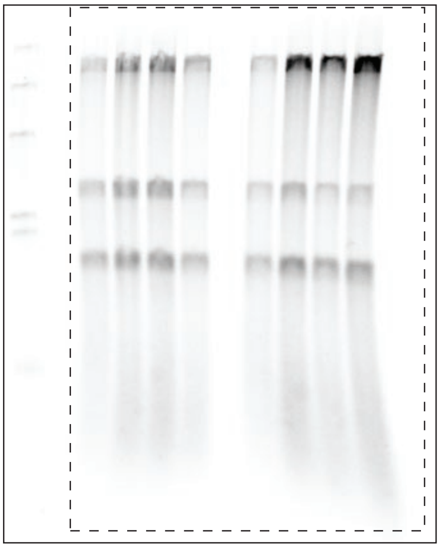

Fig S7C (left panel)

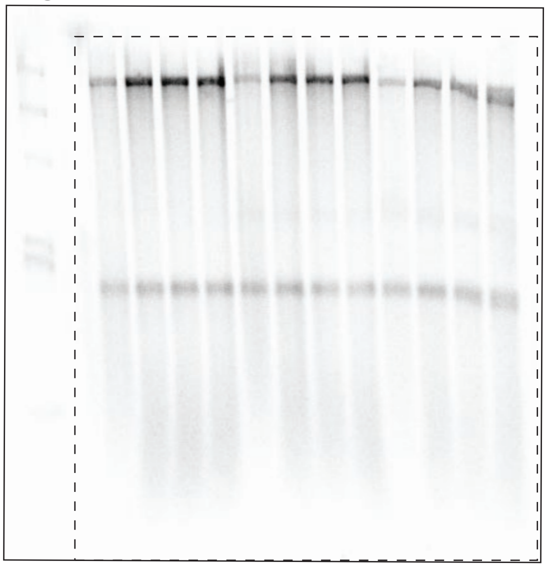

Fig S7C (right panel)

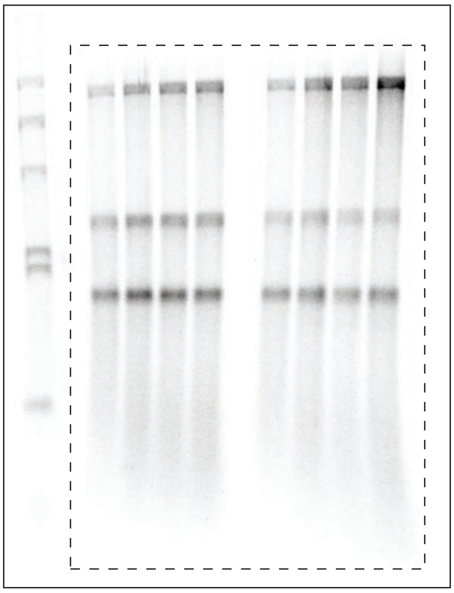

Fig S8A

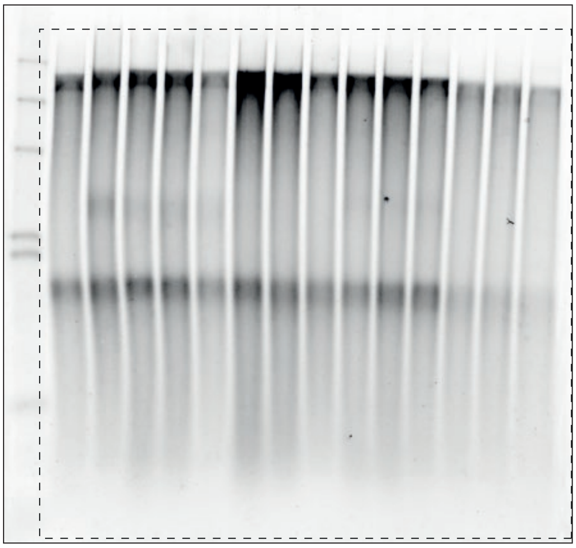

Fig S8B

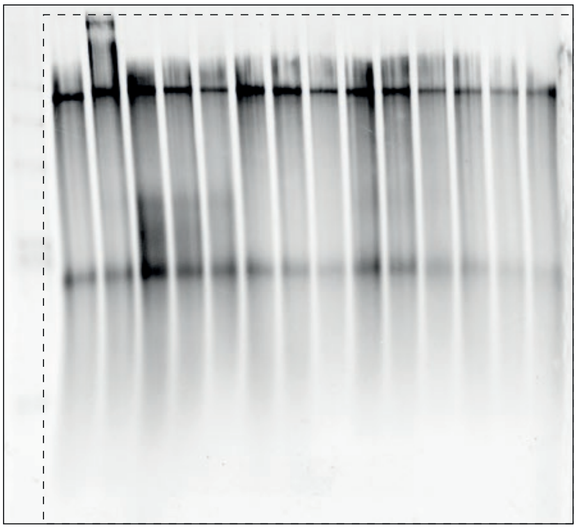

Fig S9A

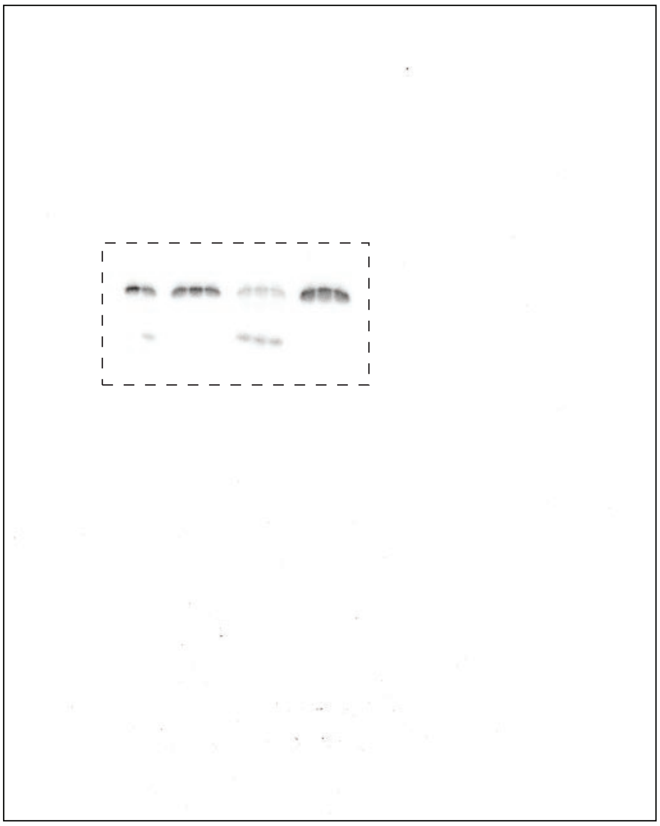

Fig S9B

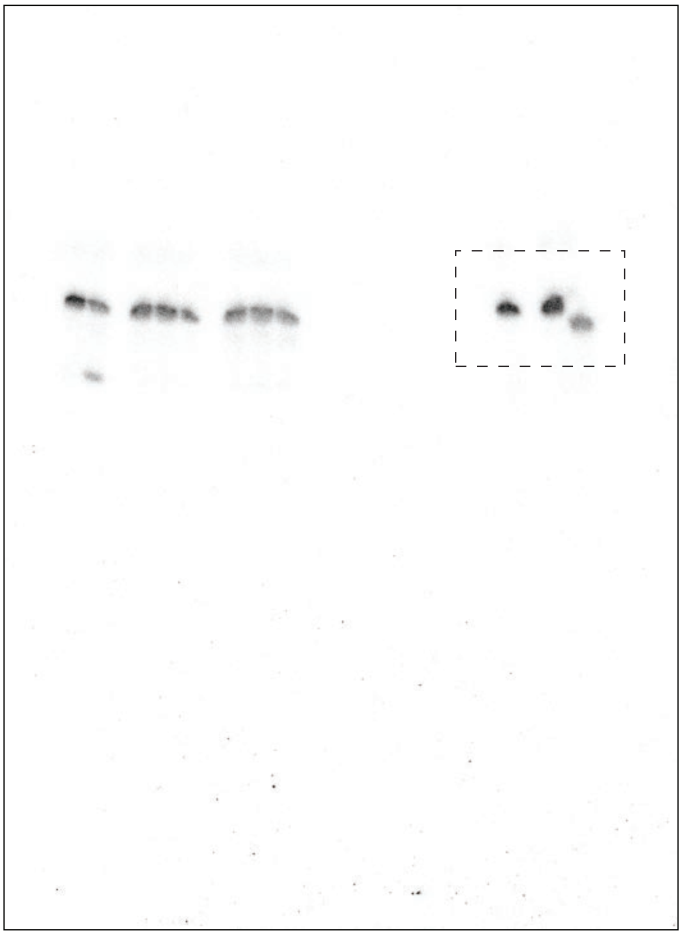

Fig S9C (left panel)

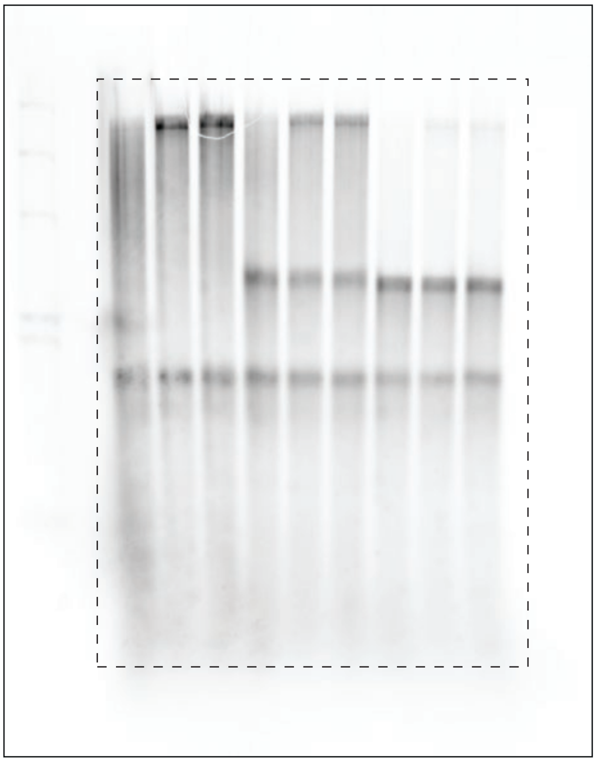

Fig S9C (right panel)

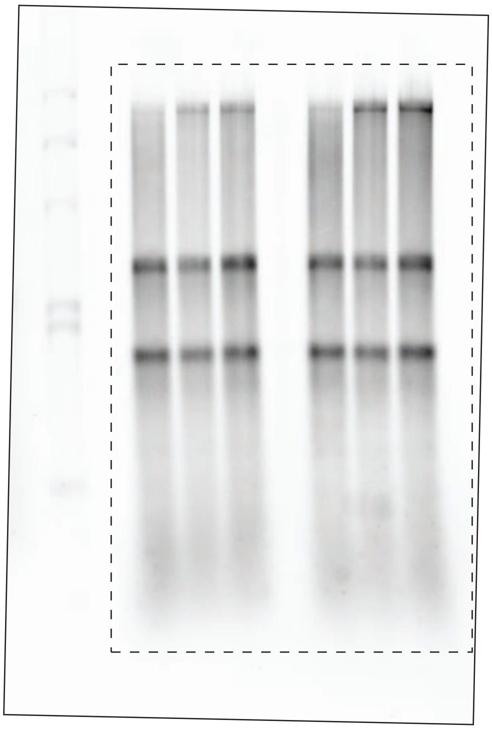

Fig S9D (left panel)

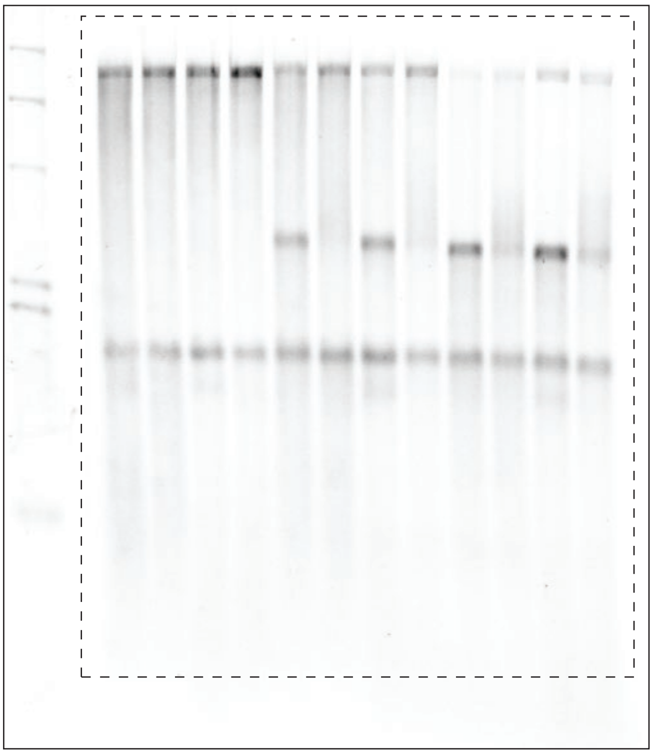

Fig S9D (right panel)

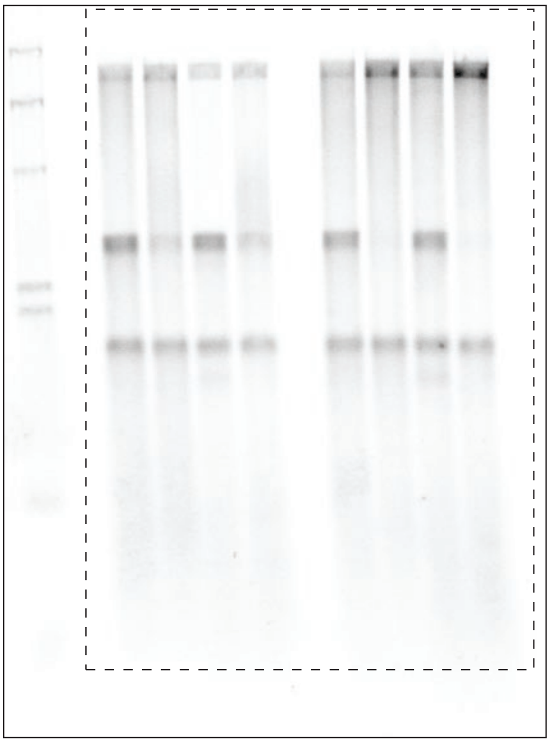

Supplement: Supplementary file 4 — Source Data [file 41467_2022_31657_MOESM4_ESM.zip › Source Data.pdf]
